# Incidence and Risk Factors of C5 Palsy following Posterior Cervical Decompression: A Systematic Review

**DOI:** 10.1371/journal.pone.0101933

**Published:** 2014-08-27

**Authors:** Yifei Gu, Peng Cao, Rui Gao, Ye Tian, Lei Liang, Ce Wang, Lili Yang, Wen Yuan

**Affiliations:** Department of Spine Surgery, Changzheng Orthopedic Hospital, Second Military Medical University, Shanghai, China; Toronto Western Hospital, Canada

## Abstract

**Background:**

C5 palsy is a serious but poorly understood complication after posterior cervical decompression that could lead to muscle weakness, brachialgia and numbness of the upper limbs. The incidence of C5 palsy varies greatly between studies. The risk factors are inconclusive and even conflicting.

**Object:**

To perform a systematic review on the incidence and risk factors of C5 palsy after posterior cervical decompression.

**Materials and Methods:**

Four databases, PubMed, Embase, Web of Science and Cochrane CENTRAL, were searched to identify eligible studies. Either a fixed- or a random-effects model was used to calculate the pooled odd ratio (RR) or standardized mean difference (SMD) with its 95% confidence interval (95%CI).

**Results:**

Of the 589 pre-recruited studies, 25 were included in this study for systematic review. The pooled incidence of C5 palsy after posterior decompression was 5.8% (95%CI: 4.4–7.2%). The incidence after open-door laminoplasty, double-door laminoplasty and laminectomy was 4.5%, 3.1% and 11.3%, respectively. The significant risk factors of C5 palsy were OPLL (OR, 2.188; 95%CI, 1.307–3.665), narrower intervertebral foramen (SMD, −0.972; 95%CI, −1.398 to −0.545), laminectomy (*vs.* open-door laminoplasty, OR, 2.988; 95%CI, 1.298–6.876), excessive spinal cord drift (SMD, 1.289, 95%CI, 0,197–2.381) and male gender (OR, 1.54; 95%CI, 1.036–2.301).

**Conclusions:**

The results of this systematic review suggest that patients with excessive spinal cord drift, preexisting intervertebral foramenal stenosis, OPLL, laminectomy and male gender are at high risk for postoperative C5 palsy, and risk-reduction options should be considered for such patients.

## Introduction

Posterior cervical decompression via laminectomy and laminoplasty is a well-recognized surgical approach for cervical myelopathy from multilevel spondylosis and/or ossification of the posterior longitudinal ligament (OPLL) [1]–[4]. Although the advantages of posterior decompression have been highly recognized in term of satisfactory surgical outcome, some postoperative problems such as axial pain, segmental instability and C5 palsy have been reported [5], [6].

Leading to muscle weakness, brachialgia and numbness of the upper limbs, C5 palsy may adds a significant burden upon patients' quality of life, and presents a financial burden on healthcare systems [7]. C5 palsy has been reported in both anterior and posterior cervical decompression, though it is more common in posterior procedures [8]. The etiology of C5 palsy has been poorly understood. Although a number of hypotheses have been suggested, the results remain inconclusive or even conflicting [9]–[11]. To obtain more precise information to help offer preoperative predicting measurements and take strategies for clinical treatment, we carried out a systematic review to clarify incidences and risk factors of C5 palsy after posterior cervical decompression.

## Methods

### Search strategies and selection criteria

We searched the electronic databases (PubMed, Embase, Web of Science, Cochrane CENTRAL) using the terms “C5 palsy”, “C5 paralysis”, “radiculopathy”, “upper limb palsy”, “upper extremity palsy”, “laminoplasty”, “laminectomy” to look for papers published in English that reported the incidence and/or risk factors and management of C5 palsy after posterior cervical spine surgery. Two reviewers (G.Y and G.R) independently evaluated the titles and abstracts of the identified papers. Only full-text articles published in English were included in this systematic review. The inclusion criteria were as follows:(1) studies consisting of 30 or more cases and focusing on C5 palsy by statistical analysis; and (2) articles referring to the posterior approach or both posterior and anterior approaches. A publication would be excluded from the systematic review if it had any of the following deficits: (1) studies without a clear definition and description of C5 palsy; (2) studies pertaining only to the anterior approach; (3)studies without defining type of surgical procedure was applied in the treatment; and (4) studies with duplicate information. If the articles were reported by the same authors from the same institute, the most recently reported paper with detailed and complete clinical data would be included.

### Data extraction

Data were recorded on a standard data extraction form, including publication details (title, authors and year), the type of study, the sample size, the type of the surgery procedures including laminectomy, open-door laminoplasty or double-door laminoplasty, the incidence, onset of C5 palsy, management and therapeutic outcome of C5 palsy, and various risk factors.

### Statistical analysis

All the included studies were divided into subgroups according to the type of surgical procedure (laminectomy, open-door laminoplasty and double-door laminoplasty) and the incidence of C5 palsy was calculated with its 95% confidence intervals (CI) for each individual study. An overall incidence of C5 palsy was calculated as a weighted average of individual summary statistics through meta-analysis, and a forest plot was obtained. Heterogeneity of effects across studies was assessed by *I* ^2^ and z test. If the z test was not significant or an *I* ^2^ value was more than 50%, the fixed-effects method was used, or otherwise the random-effects method was used. All the analyses were performed using the software Stata version 11.0(Stata Corporation, College Station, TX, USA).

## Results

### Eligible studies

**Figure 1:**
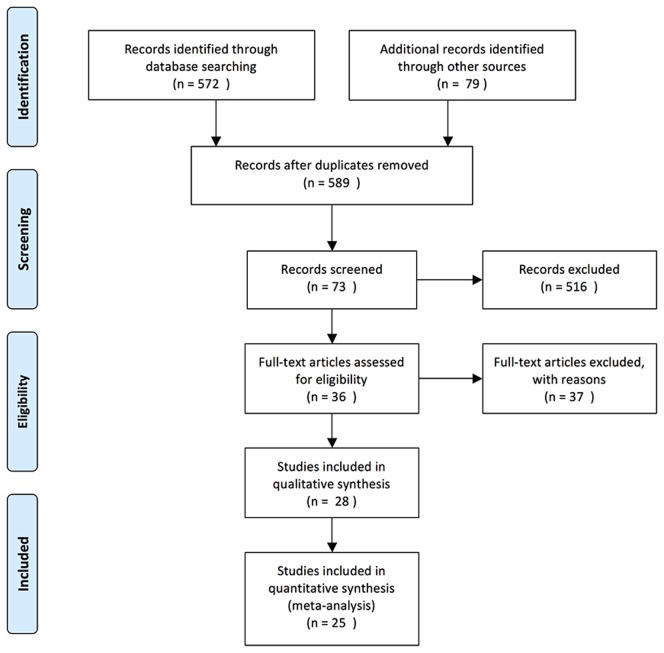
Search strategy flowchart.

A total of 589 potential studies were identified after excluding the duplications. After screening the titles and abstracts of these articles, 73 studies were recruited. After reading the full text of each study, 25 studies [2], [8], [10]–[32] were selected for this systematic review involving 5196 patients aged 40.3 to 64.0 years at the time of posterior cervical decompression surgery (Fig. 1). The detailed information about these studies is shown in Table 1. Of the 25 studies, 19 reported the incidence of C5 palsy after posterior cervical decompression, and 23 reported the potential risk factors of C5 palsy.

**Table 1:** Characteristics of the Case-Control Studies Included in the Meta-analysis.

Study	Year	No. of cases	Male/Female	Age	Surgical procedure	Cases of C5 palsy	Onset of C5 Palsy	Outcome of C5 palsy
**Lubelski D**	2013	98	NA	57.6	Laminoplasty,Laminectomy	12	NA	NA
**Yang L**	2013	141	106/35	57.97(41–75)	Open-door laminoplasty,Laminectomy	14	NA	NA
**Park JH**	2012	100	78/22	55.7	Open-door Laminoplasty, Double-door laminoplasty	7	NA	NA
**Zhang H**	2013	198	115/83	49(29–72)	Open-door laminoplasty	11	NA	All patients were treated and cured with combined dehydration by giving hormone therapy after drug treatment and rehabilitation
**Nakashima H**	2012	84	45/39	60.1(27–90)	Laminectomy	10	PO 1–9 d (mean 2.1 d)	4 patients required further surgery, others were conservatively treated with rest, muscle strength rehabilitation, and range of motion exercises in bed. Mean time to complete recovery from paralysis was 7.9 months (range 7 days–18 months).
**Nakamae T**	2012	184	130/54	64(27–89)	Open-door laminoplasty	6	NA	NA
**Radcliff KE**	2012	25	15/10	61.5(35–87)	Laminectomy	17	NA	NA
**Nassr A**	2012	221	139/82	61.7(19–87)	Open-door Laminoplasty, Laminectomy	16	PO 0–2 months	Mean time of recover to maximal improvement was 19.96 months(1–60months),3 patients had residual deficit
**Katsumi K**	2012	141	100/41	64(30–92)	Open-door laminoplasty	9	PO 0–7 d (mean 3 d)	Mean time of recovery was 3.6 months
**Xia Y**	2011	102	64/38	60.21(49–73)	Open-door laminoplasty	3	PO 1–7 d (mean 3 d)	Patients were treated by immediate management with high-dose cortical hormone therapy combined with dehydration drug therapy and physiotherapy.All recovered within 4 d–6 months
**Kaneyama S**	2010	146	108/38	64.1	Open-door Laminoplasty, Double-door laminoplasty	8	Mean PO 5.6 d	Recovered within 15 d to more than 821 d (mean 4.7 months)
**Liu T**	2010	91	54/37	40.25(20–71)	Laminectomy	21	PO 0–7 d (mean 1.7 d)	Complete recovered with in 4 weeks to 14 months
**Imagama S**	2010	1858	1096/762	62.5(36–93)	Open-door Laminoplasty, Double-door laminoplasty	43	PO 1–28 d (mean 4.7 d)	All were treated conservatively with rest, rehabilitation of muscle strength and range of movement exercises in bed, intravenous corticosteroids for two or three days, and further physiotherapy once their pain subsided. No patient with a C5 palsy needed a further operation.
**Yanase M**	2010	153	NA	65.9	Double-door laminoplasty	9	NA	NA
**Sieh KM**	2009	74	48/26	60.9(23–89)	Open-door laminoplasty	18	PO 1–7 d (mean 2.6 d)	All patients except one recovered completely, with an average of 33.1 days (1–182 days). 12 of them required simple analgesic and 6 others required anxiolytic and gabapentin for symptomatic relief.
**Takemitsu M**	2008	73	49/24	60.5(24–83)	Open-door laminoplasty and fusion	10	PO 1–7 d (mean 2.7 d)	Patients were treated conservatively. All of them fully recovered within 2 years except one patient with dural tear and suspectable direct C5 root injury at surgery. Mean time of recover duration was 9.4 months (2–24 months)
**Chen Y**	2007	49	NA	54.0	Laminectomy	9	PO 6–64 h (mean 23.6 h)	After rehabilitation therapy (functional exercises) and high-pressure oxygen therapy, 2 patients recovered within 4 to 6 postoperative months, whereas the remaining 7 required a full year before full recovery of function occurred.
**Tanaka N**	2006	62	47/15	64(32–89)	Open-door laminoplasty	3	PO 3–4 d (mean 3.3 d)	The palsy completely disappeared within 6 months in all 3 patients.
**Chiba K**	2006	80	65/15	54.4	Open-door laminoplasty	8	NA	The symptoms resolved completely within 2 years in all patients
**Komagata M**	2004	305	231/74	57(19–86)	Open-door laminoplasty	14	PO 0–28 d	Recover duration ranged from 1month to 1 year. All patients recovered completely without permanent motor palsy.
**Seichi A**	2004	114	79/35	64(33–86)	Open-door laminoplasty	9	PO 0–6 d (mean 2.11 d)	7 patients fully recovered between 3 and 23 months, 2 patients partially recovered within follow-up.
**Minoda Y**	2003	45	35/10	58.2(36–80)	Double-door laminoplasty	4	PO 0–7 d (mean 2.0 d)	Complete recovery of these palsies was confirmed between 4 weeks and 17 months after development of the palsy.
**Fan D**	2002	200	131/69	59.6(28–89)	Laminectomy	8	NA	2 patients underwent a additional anterior cervical discectomy.The mean recovery duration of all the patients was 5 months (24 h–2 year)
**Uematsu Y**	1998	365	NA	NA	Open-door laminoplasty	20	PO 6 h–20 d	All patients were treated by conservative therapy alone.18 patients recovered completely within 2 d to 26 months,2 patients recovered up to the point of MMT 4 by PO 4 years.
**Dai LY**	1998	287	NA	NA	Laminectomy	37	PO 4 h–6 d (mean 15 h)	The mean time for recovery was 5.4 months (range from two weeks to three years)

### Incidence of C5 palsy

**Figure 2:**
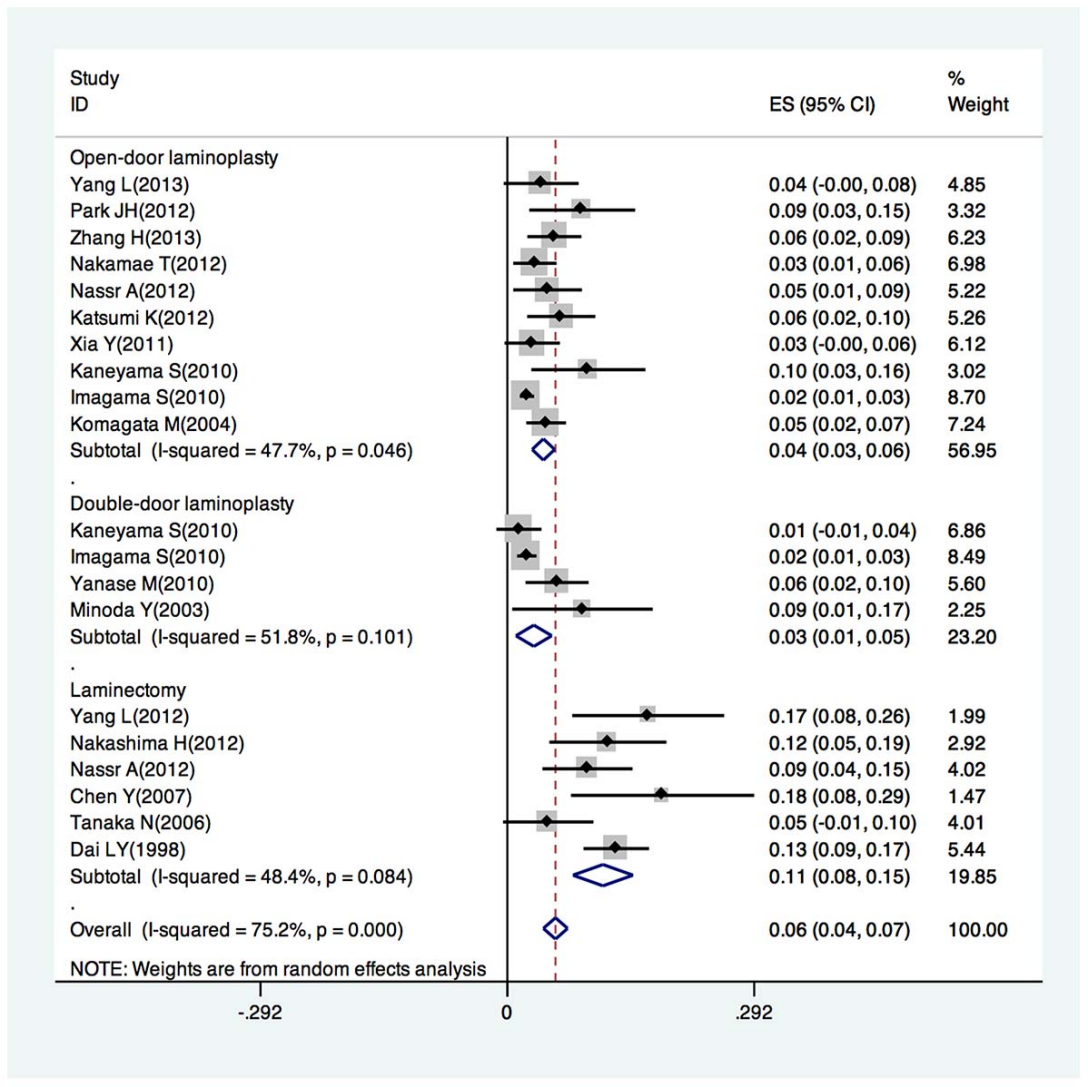
Forest plot for incidence of posterior C5 palsy in patients underwent open-door laminoplasty, double-door laminoplasty, laminectomy, repectively.

The incidence of C5 palsy was reported in 19 studies. The repoted incidence of C5 palsy after posterior decompression ranged from 1.4% to 18.4%. A pooled incidence was 5.9% (95%CI:4.5–7.4%), with a statistically significant heterogeneity between the studies(I^2^ = 75.2%,P<0.001)(Fig. 2).The incidence varied significantly across studies depending on the type of surgical procedure.

Ten studies reported the incidence of C5 palsy ranging from 2.3% to 9.6% in patients who underwent open-door laminoplasty, with a pooled incidence of 4.3% (95% CI:2.9–5.6%, I^2^ = 47.7%,P = 0.046).

Four studies reported the incidence of C5 palsy ranging from 1.4% to 8.9% in patients who underwent double-door laminoplasty. with a pooled incidence of 3.1% (95% CI:1.0–5.3%). There was no statistically significant heterogeneity between the studies (I^2^ = 51.8%,P = 0.101).

Six studies reported the incidence of C5 palsy ranging from 4.8% to 18.4% in patientss who underwent laminectomy with a pooled incidence of 11.3% (95% CI:7.8–14.9%). There was no statistically significant heterogeneity between the studies (I^2^ = 48.4%, P = 0.084).

The period from surgery to the onset of C5 palsy can varied from immediately to 2 months after surgery. Most patients in these studies recovered within a week to several months after conservative treatments including rest, muscle strength rehabilitation, hyperbaric oxygen therapy and/or immediate drug therapy including high-dose cortical hormone therapy combined with dehydration therapy. One study reported 2 patients who recovered up to the point of MMT 4 after 4 years post-operation. Two studies reported 6 patients who suffered from residual deficits until the end of follow-up. Two studies reported a total of 6 patients who required a further surgery to ease the symptoms.

### Risk factors of C5 palsy

**Figure 3:**
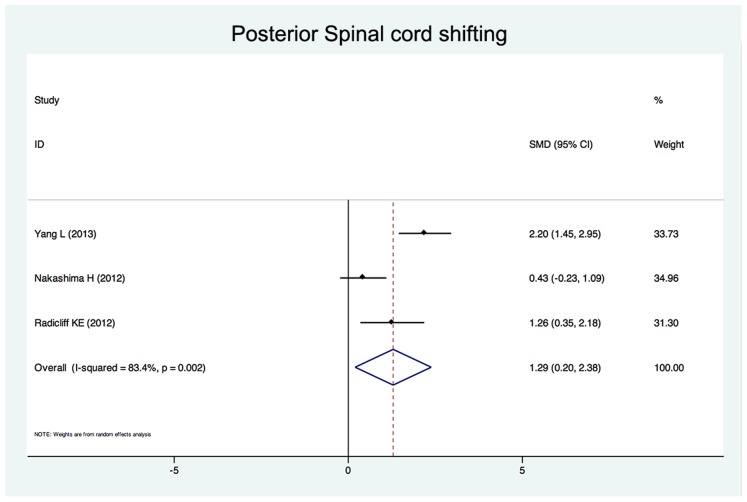
Forest plots for posterior spinal cord shifting. The width of the horizontal line represents the 95% confidence interval (CI) of the individual studies, and the square proportional represents the weight of each study. The diamond represents the pooled standardized mean difference (SMD) and 95% CI.

Twenty-five studies reported the risk factors of C5 palsy after posterior decompression. The main results are shown in Table 2. Significant risk factors were OPLL (OR, 2.188; 95%CI, 1.307 to 3.665), narrower intervertebral foramen (SMD, −0.972; 95%CI, −1.398 to −0.545), laminectomy (*vs.* open-door laminoplasty, OR, 2.988; 95%CI, 1.298 to 6.876), excessive spinal cord drift (SMD, 1.289, 95%CI, 0,197 to 2.381) and male gender (OR, 1.54; 95%CI, 1.036 to 2.301). Forest plots of these 5 significant results are shown in Fig. 3 to Fig. 7. Age, preoperative Japanese Orthopeadic association (JOA) score, pre- and post-operative lordotic cervical angle, double-door laminoplasty (*vs.* open-door laminoplasty) and T2 high-signal lesion of C3–C5 on MRI proved to be no significant (P>0.05).

**Figure 4:**
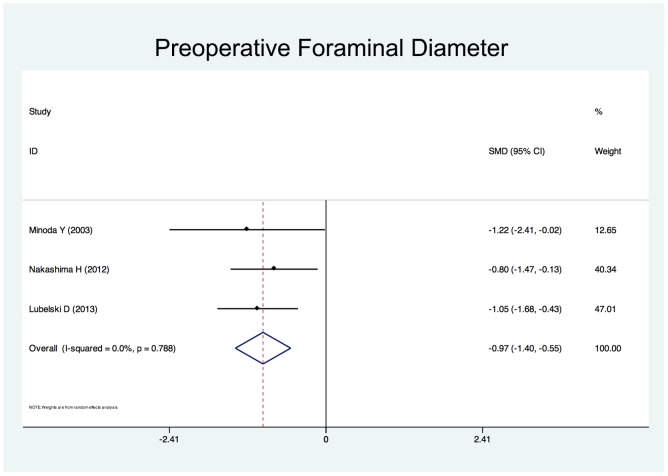
Forest plots for intervertebral foraminal diameter. The width of the horizontal line represents the 95% confidence interval (CI) of the individual studies, and the square proportional represents the weight of each study. The diamond represents the pooled standardized mean difference (SMD) and 95% CI.

**Figure 5:**
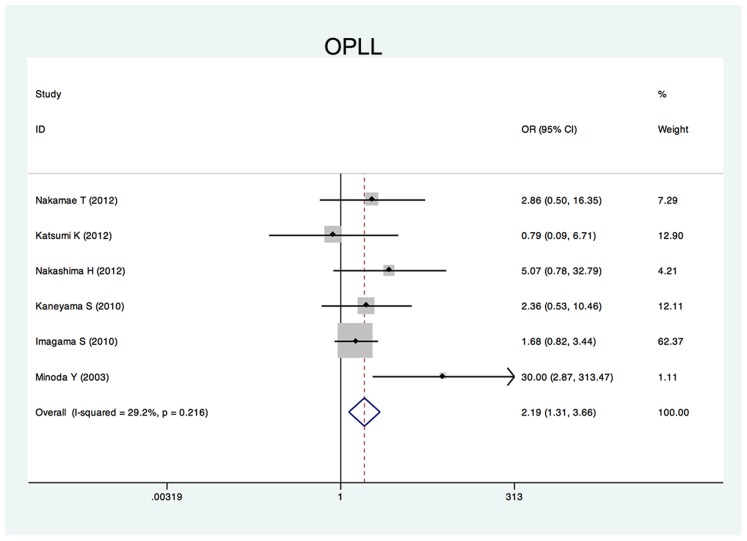
Forest plots for ossification of posterior longitudinal ligament. The width of the horizontal line represents the 95% confidence interval (CI) of the individual studies, and the square proportional represents the weight of each study. The diamond represents the pooled odds ratio (OR) and 95% CI.

**Figure 6:**
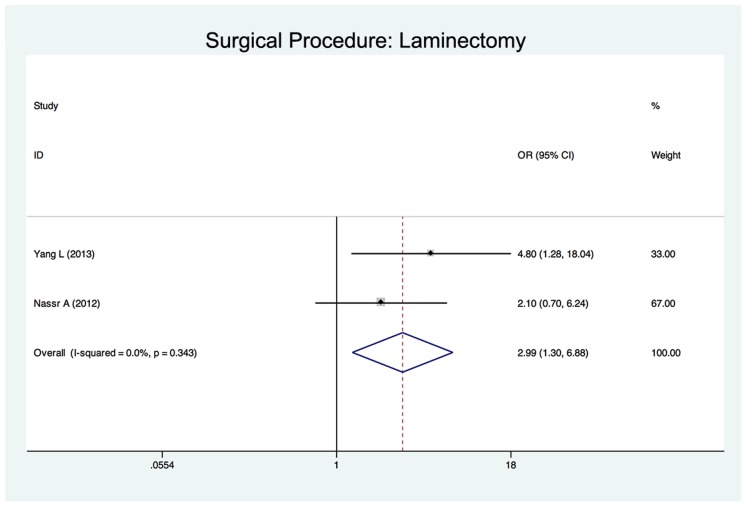
Forest plots for surgical procedure. The width of the horizontal line represents the 95% confidence interval (CI) of the individual studies, and the square proportional represents the weight of each study. The diamond represents the pooled odds ratio (OR) and 95% CI.

**Figure 7:**
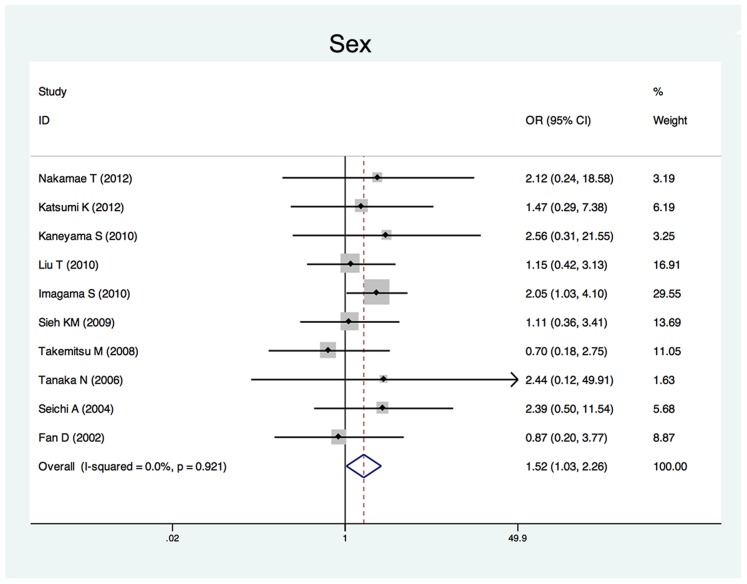
Forest plots for sex. The width of the horizontal line represents the 95% confidence interval (CI) of the individual studies, and the square proportional represents the weight of each study. The diamond represents the pooled odds ratio (OR) and 95% CI.

**Table 2:** Summary of Odds Ratios (ORs) and Standardized Mean Differences (SMDs) and 95% Confidence Intervals (CIs) of Significant Risk Factors for postoperative C5 palsy.

Risk factors	OR or SMD	95% CI	P Value for heterogeneity
Significant factors			
OPLL	2.188*	1.307–3.665	0.216
Preoperative foraminal diameter	−0.972†	−1.398 to −0.545	0.788
Laminectomy (vs. open-door laminoplasty)	2.988*	1.298–6.876	0.343
Posterior spinal cord shifting	1.289†	0.197–2.381	0.002
Male	1.544*	1.036–2.301	0.921
Insignificant factors			
Preoperative JOA score	1.319†	−0.207 to 2.845	0.000
Post-operative cervical lordotic angle	0.87†	−0.015 to 1.755	0.000
Pre-operative cervical lordotic angle	−1.111†	−2.503 to 0.281	0.000
Double-door laminoplasty (vs. open-door laminoplasty)	0.75*	0.427 to 1.317	0.108
T2 high-signal lesion on MRI	0.748*	0.408 to 1.37	0.539

*OR.

† SMD.

## Discussion

A number of studies have demonstrated the occurrence of C5 palsy after posterior cervical decompression. Although various mechanisms underlying this serious complication have been proposed, controversies still exist. Sakaura et al [33] summarized the some most possible pathologic mechanisms of C5 palsy, including the intraoperative nerve root injury, nerve root traction, spinal cord ischemia, segmental spinal cord disorder and reperfusion injury of the spinal cord.

The aim of the present systematic review was to evaluate the incidence and risk factors of C5 palsy after posterior cervical decompression. It was found that the pooled incidence of C5 palsy after posterior decompression is 5.8%. The incidences reported in different studies are highly variable, ranging from 1.4% to 18.4%. Such a large variation can be explained by the difference in the type of surgical procedures applied between studies. Three main types of surgical procedures were employed in these studies, and the pooled incidence of each procedure was significantly discrepant. The heterogeneity decreased when the studies were divided into three subgroups according to the surgical procedure. Even when the surgical procedure was the similar, subtle differences in specific techniques existed. Whether a foraminotomy was performed or not, the extent of decompression, the open-angle of lamina and the method of internal fixation may all contribute to the great variation in the incidence of C5 palsy [13], [14], [25]. In addition, there were differences in how C5 palsy was defined between the studies. For instance, Imagama et al [22] defined C5 palsy as a postoperative 0 to 2 manual muscle test (MMT) grade in the deltoid, with or without involvement of the biceps muscle without loss of strength in other muscles. Nakashima et al [15] defined C5 palsy as as postoperative deterioration by ≥1 MMT grades in the deltoid, with or without involvement of the biceps muscle. Nassr et al [8] defined C5 palsy as loss of motor strength in the deltoid and/or biceps brachii, sensory deficit in the C5 distribution, or increased pain in the C5 distribution as compared with the preoperative status. Nakamae et al [16] defined C5 palsy as postoperative motor palsy of the deltoid and biceps muscles in the upper extremity by ≥1 grades in the manual muscle test (MMT) without sensory disturbance. These results highlight the need of a standard definition of C5 palsy in future studies.

Although the occurrence of C5 palsy after posterior cervical decompression has been reported in many studies, its detailed mechanism remains poorly understood. There have been several hypotheses regarding the etiology of C5 palsy, including direct injury to nerve root during the operation [10], tethering of the nerve root [9], segmental spinal cord disorder [30], and ischemia/reperfusion injury of the spinal cord [34], but none of these hypotheses have been completely established. The result of this study showed that a narrower intervertebral foramen, excessive spinal cord drift, OPLL, laminectomy and the male gender are risk factors of C5 palsy after posterior cervical decompression.

Nerve root traction may be caused by posterior drift of the spinal cord after posterior cervical decompression, so called “tethering effect” was considered one of the most acceptable pathologic mechanisms of C5 pasly [9]. Shiozaki et al [35] found a significant posterior shift of the spinal cord on MRI 24 hours after posterior decompression. The maximum posterior shift occurred at the C5 vertebral level because C5 is the apex of cervical lordosis. In addition, the superior articular process of C5 protrudes in a more anterior direction and root of C5 are shorter as compared with other levels, the posterior shift might creat a tension on C5 nerve root, causing C5 palsy [22]. Three studies enrolled in this systematic review showed that the posterior shift in patients with C5 palsy was significantly larger than that in patients without palsy. For this reason, some scholars suggested a limited decompression to avoid excessive posterior shifting of the spinal cord [13], [36].

Preexisting foraminal stenosis has been suggested to be associated with C5 palsy in several studies. Imagama et al [22] reviewed 1858 patients who had undergone a cervical laminoplasty. They found the width of the C5 intervertebral foramen (both on the palsy side and normal sides) were significantly smaller, and anterior protrusion of the C5 superior articular process were significantly greater in patients with C5 palsy. Katsumi et al [18] reported a significant difference in preexisting C4/5 foraminal stenosis in patients with C5 palsy. Our systematic review also suggests preexisting foraminal stenosis as a risk factor of C5 palsy. Several studies have recommend prophylactic foraminotomy to prevent C5 palsy. Komagata et al [29] reported that prophylactic bilateral partial foraminotomy could reduce the incidence of C5 palsy after open-door laminoplasty from 4.0% to 0.6%. Yanase et al [23] suggested foraminotomy in those patients with narrowed foramina after pre or intraoperative electrophysiological tests.

Several studies have reported a higher incidence of C5 palsy in patients with OPLL [20], [22], [31], presumably because the ossified hypertrophic posterior longitudinal ligament increased the spinal cord shifting and tethering effect on the C5 nerve root [26]. Our systematic review showed that OPLL was a significant risk factor of postoperative C5 palsy compared with cervical spondylotic myelopathy and other cervical degeneration diseases.

Two papers enrolled in our study compared patients who underwent laminectomy with those who underwent laminoplasty [8], [13]. The results showed that the incidence of C5 palsy was significant higher in laminectomy group and suggested laminectomy as a significant risk factor. That may be because the laminectomy removes the intact posterior arch of the vertebra, thus providing an excessive space for the spinal cord to shift posteriorly. Radcliff et al [17] reported that a narrower laminectomy trough width could prevent the spinal cord from shifting excessively, thus reducing the incidence of C5 palsy. Three studies enrolled in this systematic review compared the incidence of C5 palsy in open-door laminoplasty and double-door laminoplasty [2], [20], [22]. One study showed that in patients underwent open-door laminoplasty, especially in those with OPLL, the spinal cord was prone to rotate due to asymmetrical decompression, resulting in the tethering nerve root on the open side [20]. However, a large-sample and multicentre study [22] reported no significant difference in the incidence of C5 palsy between patients who underwent open-door laminoplasty and those who underwent double-door laminoplasty. Our systematic review showed that open-door laminoplasty was not a significant risk factor of C5 palsy. There is no study comparing laminectomy with double-door laminoplasty.

There are controversies over whether intraoperatively correction of the cervical lordotic alignment has an effect on the occurrence of C5 palsy. Takemitsu et al [25] reported that the cervical curvature of patients who developed C5 palsy underwent a significant change, supposing that cervical alignment correction by posterior instrumention might cause iatrogenic foraminal stenosis and excessive posteriorly shifting of the spinal cord. However, most other authors suggested that there was no significant correlation between sagittal alignment and posterior shifting of the spinal cord [36], [37]. Our systematic review also showed that change in cervical alignment is not a significant risk factor of C5 palsy.

An alternative hypothesis is that C5 palsy might be caused by a spinal cord disorder [34]. However, this systematic review showed that neither preoperative JOA score nor T2 high-signal intensity zone in the spinal cord on MRI imaging is a significant risk factor. Some authors hypothesized that C5 may be caused by intra-operative injury of spinal cord or nerve root [22], [38]. Imagama et al [22] supposed that the nerve root was probably damaged at the time of operation by the heat generated by the high-speed drill, which would make it an iatrogenic injury. Takenaka et al [39] suggested using cooled irrigation saline during bone drilling during laminoplasty to prevent C5 palsy. However, more convincing evidence is needed to support this hypothesis in further studies. Nakamae et al [16] found that postoperative C5 palsy after cervical laminoplasty occurred in cases without significant abnormal findings during intra-operative monitoring.

Some other risk factors were also reported in individual studies. Xia et al [19] reported that in open-door laminoplasty, patients with a relative lateral through on the hinge side .were more susceptible to C5 palsy. Zhang et al [14] considered that the lamina open angle in laminoplasty should be maintained between 15°∼30°, or otherwise the risk of postoperative C5 palsy may increase.Radcliff et al [17] found a wider laminectomy at C5 and an increased diameter of the spinal canal were associated with an increased risk of C5 palsy. Preoperative compression at C3 level preoperatively [31] and larger anterior protrusion of C5 superior articular process [22] were also mentioned to be risk factors. Although the dependability of these risk factors needs to be confirmed in further studies, they may provide some valuable suggestion in the studies of C5 palsy after cervical decompression surgery.

## Conclusions

C5 palsy is a severe complication associated with posterior cervical decompression. The incidence of C5 palsy varies significantly between studies. Excessive spinal cord shifting, preexisting intervertebral foramenal stenosis, OPLL, laminectomy and male gender are risk factors of postoperative C5 palsy. These findings may be constructive to clinical surgeons to reduce the incidence of C5 palsy by setting up preoperative predictive measurements and take appropriate surgical strategies on the basis of the individuality of patients.

## Supporting Information

Checklist S1 **PRISMA checklist.**
(DOC)
